# A clinically deployable deep learning model for automated stroke risk stratification in carotid atherosclerotic plaque

**DOI:** 10.3389/fmed.2026.1764968

**Published:** 2026-04-21

**Authors:** Yafei Gao, Hao Wang, Dingwen Zhou, Peipei Mai, Xiaona Li, Panpan Li, Yongxin Li, Hua Wang

**Affiliations:** 1 Department of Ultrasonography, Luoyang Central Hospital Affiliated to Zhengzhou University, Luoyang, Henan, China; 2 School of Automation and Intelligence, Beijing Jiaotong University, Beijing, China

**Keywords:** carotid plaque, deep learning, diagnostic accuracy, risk prediction, stroke, ultrasound images

## Abstract

**Background:**

Carotid ultrasound is widely utilized for early risk screening of ischemic stroke. However, the accuracy and reproducibility of assessing plaque vulnerability-related features remain constrained by physicians’ subjective interpretation, underscoring an urgent need to achieve precise and objective assessment of these features through intelligent quantification.

**Objective:**

This study aims to develop and compare deep learning (DL) and conventional machine learning (ML) models based on carotid plaque ultrasound images, so as to identify the optimal clinically applicable algorithm for precise plaque assessment and risk prediction.

**Methods:**

In this retrospective cohort study, 666 patient’s carotid plaque ultrasound images (299 stroke patients; 367 non-stroke controls) collected between 2021 and 2025 were analyzed. Five convolutional neural networks (CNNs, e.g., ResNet-50) and two conventional machine learning (ML) classifiers [support vector machine (SVM), logistic regression (LR)] were trained on region-of-interest annotated plaque images using an 8:2 training-to-validation split. The area under the receiver operating characteristic curve (AUC) served as the primary performance metric, supplemented by accuracy, sensitivity, and specificity as secondary evaluation indices. The stroke risk prediction efficacy of the optimal DL model was subsequently compared with that of the ML models.

**Results:**

Among five DL models evaluated, ResNet-50 demonstrated optimal diagnostic performance for stroke risk stratification in carotid plaque patients, achieving an AUC of 0.982 (accuracy: 0.925, sensitivity: 0.964, specificity: 0.897) on the independent test set. For traditional ML models, LR marginally outperformed SVM (AUC: 0.885 vs. 0.861), though without statistical significance (DeLong test: *z* = 0.591, *p* = 0.554). Critically, the best-performing DL model (ResNet-50) exhibited a 9.7% improvement in AUC over the top ML model (0.982 vs. 0.885), with consistently superior accuracy, sensitivity, and specificity across all metrics.

**Conclusion:**

This study validates the superiority of the ultrasound image-based lightweight deep learning model (ResNet-50) in predicting stroke risk in patients with carotid plaques, making it a preferred clinical diagnostic tool.

## Introduction

Stroke stands as the third leading cause of death globally and a primary contributor to long-term disability, with an annual global incidence exceeding 6 million cases (1, 2). The development of cerebrovascular events is strongly associated with carotid atherosclerotic plaques, particularly unstable ones (3–5). Notably, rupture of unstable carotid plaques is directly attributable to up to one-quarter of all strokes (6–8). This substantial burden underscores the critical need for early and precise risk stratification of carotid plaques. Implementing individualized prevention and treatment strategies based on such stratification offers a potent means to disrupt the pathological cascade leading to ischemic stroke, thereby significantly reducing associated morbidity and mortality—a paramount clinical objective.

Ultrasound remains the first-line screening tool for carotid atherosclerosis due to its non-invasiveness, real-time capability, and cost-effectiveness (9). First, conventional assessment methods heavily rely on physicians’ subjective interpretation of morphological features (such as surface ulceration, echogenicity, and calcification patterns), which demonstrates poor inter-observer agreement and limited reproducibility. Second, although traditional ML models have been used to enhance objectivity, they are inherently constrained by their dependence on manual feature engineering—a process that is not only time-consuming and expertise-dependent but may also fail to capture the complex, sub-visual texture characteristics indicative of plaque vulnerability, thereby limiting their predictive performance in meeting the demands of clinical precision.

DL has emerged as a transformative force in medical image analysis, offering a powerful solution to address the limitations of traditional carotid plaque characterization. Unlike traditional ML methods, which rely on cumbersome and subjective manual feature engineering, DL models can directly learn and extract complex subvisual features from raw image data in an end-to-end manner (10–12). This capability enables the identification of discriminative patterns that lie beyond both the perceptual limits of human vision and the representational scope of predefined radiomic formulas, such as subtle textural heterogeneity indicative of intraplaque hemorrhage or microcalcification clusters. Consequently, numerous studies have reported that deep learning demonstrates superior performance in both plaque classification and vulnerability assessment, laying the foundation for developing automated, high-precision stroke risk stratification models (13–15). Among the various deep learning algorithms, Convolutional Neural Networks (CNNs)—notably architectures like VGG-16, ResNet and their derivatives—have demonstrated superior efficacy in image feature classification (16–18).

Although both ML and DL have been independently applied to predict stroke risk in patients with carotid plaque, a critical evidence gap persists: the absence of rigorous, direct comparisons between these paradigms within identical patient cohorts and unified validation frameworks. Consequently, whether DL provides a definitive and clinically significant performance advantage over well-constructed traditional ML models for this specific predictive task remains an unresolved question.

Therefore, this study aims to conduct a comprehensive, head-to-head comparison of state-of-the-art deep learning (DL) models versus robust traditional machine learning (ML) models for predicting early ischemic stroke risk in patients with carotid atherosclerotic plaque, using ultrasound images. We will develop and validate a radiomics-based ML classifier against an end-to-end DL convolutional neural network (CNN) within a single cohort. By evaluating their performance under identical conditions, this research seeks to identify the most accurate and clinically translatable AI paradigm to optimize stroke risk stratification, enabling early identification of high-risk individuals for targeted preventive interventions.

## Materials and methods

### Objects

**Figure 1:**
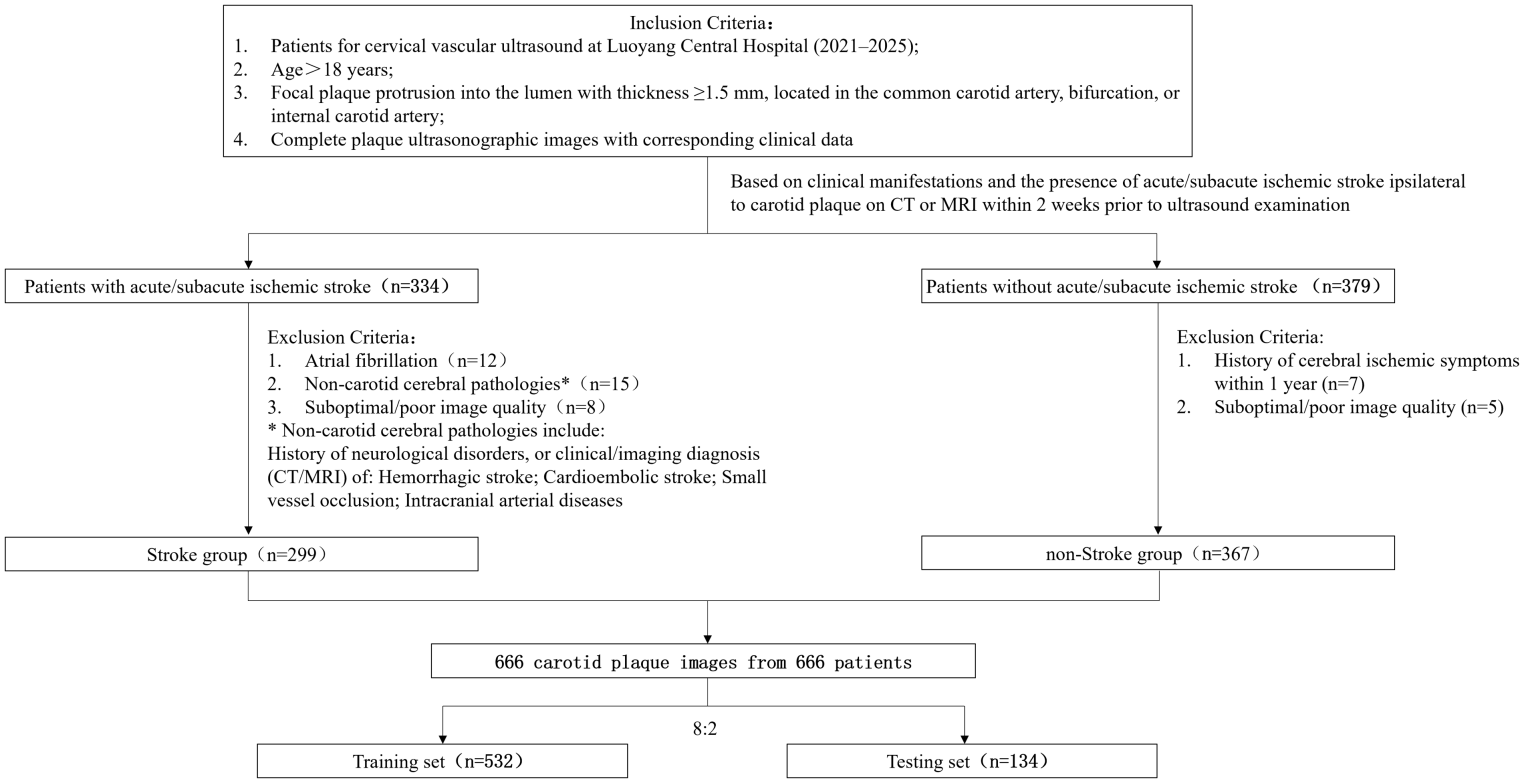
The flow diagram of recruitment and grouping of research objects.

The study retrospectively collected data from patients who underwent carotid ultrasound examination at Luoyang Central Hospital Affiliated to Zhengzhou University from 2021 to 2025. Inclusion criteria were: (1) age >18 years; (2) Focal plaque protrusion into the arterial lumen with thickness ≥1.5 mm, localized in the common carotid artery (CCA), bifurcation, or internal carotid artery (ICA); (3) availability of complete plaque ultrasound images and clinical data. Based on the presence of clinical symptoms 2 weeks prior to US examination and/or whether there is acute/subacute anterior circulation ischemic stroke on the same side of the carotid artery plaque on multislice spiral computed tomography (CT)/magnetic resonance imaging (MRI) (19, 20), patients were classified as stroke group and non-stroke group. Exclusion criteria for the stroke group were: (1) atrial fibrillation; (2) non-carotid etiologies of cerebral abnormalities (including hemorrhagic stroke, cardioembolic infarction, small vessel occlusion, or intracranial arteriopathy diagnosed via neurological history, clinical symptoms, and/or CT/MRI); (3) poor-quality or non-diagnostic images. Exclusion criteria for the non-stroke group were: (1) history of cerebral ischemic symptoms within the preceding year; (2) poor-quality or non-diagnostic images. Ultimately, the study enrolled 299 patients in the stroke group and 367 patients in the non-stroke group (Figure 1).

The study was conducted in compliance with the Declaration of Helsinki and approved by the Institutional Review Board.

### Ultrasound images acquisition

All carotid ultrasound examinations were performed by certified sonographers with 5–10 years of vascular ultrasound experience using a Toshiba Aplio 500 ultrasound system (Toshiba Medical Systems, Tokyo, Japan) equipped with a 4.2-14 MHz linear array transducer.

Patients were positioned in a semi-recumbent position with slight neck extension and contralateral rotation. Bilateral common carotid arteries (CCAs), bifurcations, and internal carotid arteries (ICAs) were systematically scanned in longitudinal and transverse planes. The largest plaque on the symptomatic side underwent multiplanar assessment to document: Surface morphology; Internal echogenicity; Presence of lipid core. Plaques were classified by echogenicity into three types (21): (1) Hypoechoic/Isoechoic; (2) Heterogeneous; (3) Hyperechoic. Surface morphology was graded per DeGray (22) classification: (1) Smooth: (2) Regular contour; (3) Ulcerated: Depression with width & depth ≥2 mm; Irregular: Non-ulcerated uneven surface. Calcification patterns were categorized as: Morphology: (a) Absent; (b) Microcalcifications/punctate; (c) Macrocalcifications/dense. Distribution: (a) Superficial; (b) Basal; (c) Intraplaque. Ultrasound static images were exported in JPG format for subsequent analysis.

### Deep learning model development

Ultrasound images in JPG format were imported into the MedAI Darwin learning platform.^1^ Using the platform’s tools, the plaque was delineated as the region of interest (ROI). The annotation process was performed by an experienced vascular ultrasound specialist, and any disagreements were resolved through consensus discussion. Strict adherence to clinical ultrasound diagnostic standards and relevant literature guidelines ensured that the ROI accurately reflected the target plaque region. The dataset included 666 images with annotated ROIs. The annotated data were randomly divided into a training set (*n* = 532) and a testing set (*n* = 134) in an 8:2 ratio. Before model training, the input ultrasound images were first subjected to preprocessing such as noise reduction, gain normalization, and artifact removal, endowing the model with certain anti-interference ability and robustness. Following this, preprocessing operations such as data augmentation and normalization were performed on the input ROI sub-images, including random flipping, image transposition, and pixel value normalization (23).

**Figure 2:**
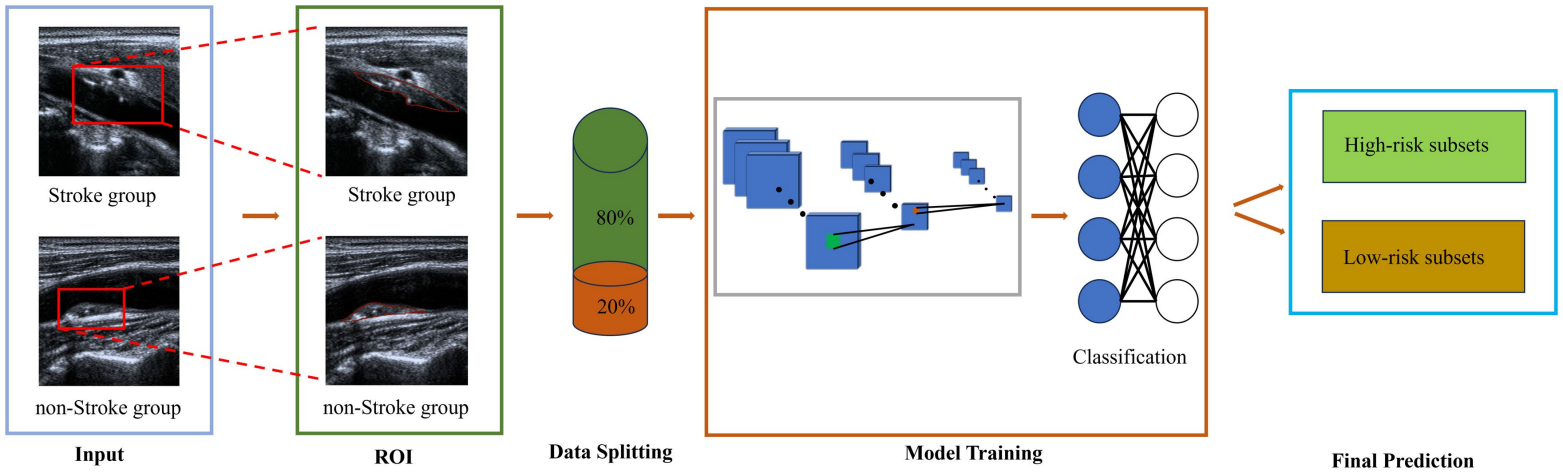
Schematic diagram of DL models in predicting the stroke risk.

Five DL architectures were employed: ResNet-50, SE-ResNet-50, SE-ResNeXt-50, DenseNet-121 and InceptionResNet-V2. These models were trained separately on plaque images to perform stroke risk prediction and stratification predict stroke risk and perform risk stratification in patients with carotid artery plaques. Model performance was evaluated using standard diagnostic metrics, including the area under the receiver operating characteristic (ROC) curve (AUC), accuracy, sensitivity, specificity, positive predictive value (PPV), and negative predictive value (NPV). Receiver operating characteristic (ROC) curves were generated to visualize and compare the classification performance of the models. Figure 2 illustrates the overall experimental workflow.

### Machine learning model development

Radiomics feature extraction was performed using the PyRadiomics package (version 3.1.0) integrated within the MedAI Darwin platform.^2^ Prior to feature extraction, ultrasound images underwent intensity normalization to standardize acquisition parameters. A total of 1,125 radiomics features were subsequently extracted from each manually delineated ROI. The extracted radiomics features comprised four categories: (1) 2D shape features; (2) First-order intensity features; (3) Higher-order texture features (derived from original images); (4) Wavelet-based features (extracted from images transformed via wavelet decomposition). The 1,125 radiomics features underwent *Z*-score normalization, followed by a three-tiered feature selection pipeline: (1) univariate screening via independent samples *t*-test retaining features with *p* < 0.05; (2) redundancy reduction using Pearson/Spearman correlation analysis (features with |*r*| > 0.9 eliminated); and (3) dimensionality reduction through least absolute shrinkage and selection operator (LASSO) regression with 10-fold cross-validation to identify the optimal *λ* hyperparameter minimizing mean squared error (MSE), ultimately yielding the optimal feature subset.

**Figure 3:**
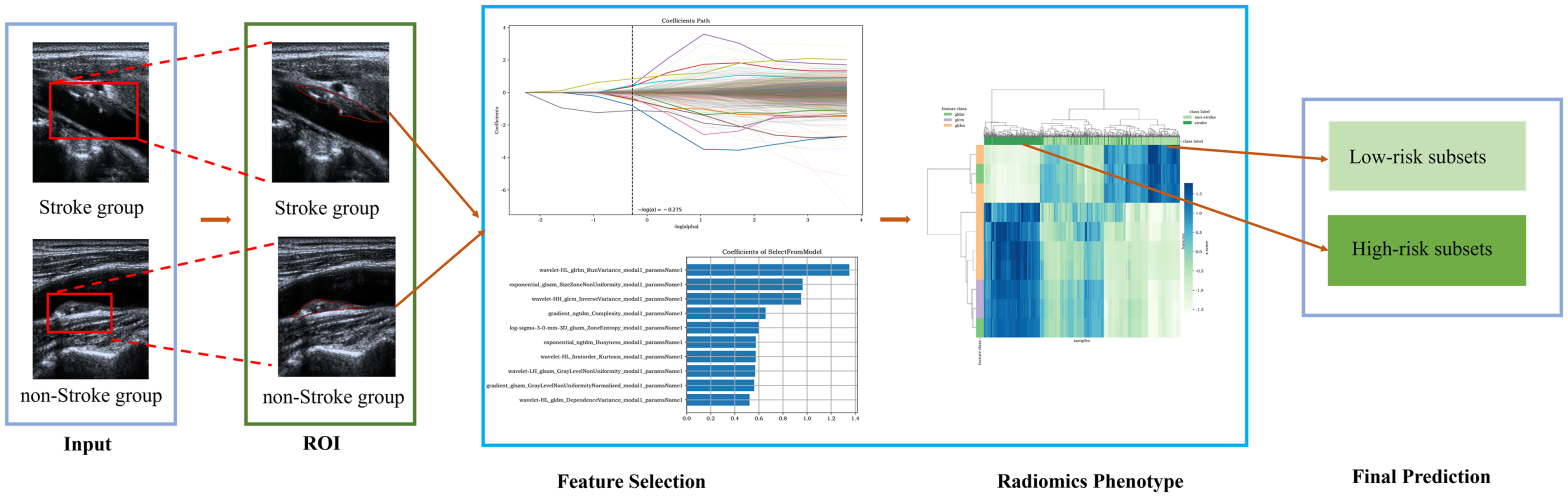
Schematic diagram of ML models in predicting the stroke risk.

Following feature selection, two supervised machine learning classifiers—LR and SVM—were developed to construct classification models. Model discrimination performance was evaluated using receiver operating characteristic (ROC) curves and area under the curve (AUC), while calibration was assessed via calibration curves and Hosmer-Lemeshow goodness-of-fit tests. Comparative validation across models in an independent cohort enabled identification of the optimal radiomics model for stroke risk prediction in patients with carotid artery plaques. All predictive models underwent rigorous validation in an independent cohort, with comparative performance assessment identifying the optimal radiomics model for stroke risk stratification in patients with carotid artery plaques. The complete experimental workflow is shown in Figure 3.

### Statistical analysis

Statistical analyses were conducted using SPSS software (version 26.0; IBM Corp). Continuous variables were expressed as mean ± standard deviation (SD) and compared using independent samples *t*-tests. Categorical variables were presented as counts and percentages and analyzed using chi-square tests. The predictive performance of the model was evaluated by plotting receiver operating characteristic (ROC) curves and calculating the area under the curve (AUC). Comparisons of AUC values between models were performed using *z*-tests. All statistical tests were two-tailed, and a *p*-value <0.05 was considered statistically significant.

## Results

### Baseline characteristics

Our cohort comprised 666 carotid ultrasound images. The baseline characteristics of this cohort included 400 male and 266 female patients with a mean age of 66.15 ± 9.87 years. The Low-density lipoprotein (LDL) cholesterol was 2.37 ± 0.85 mmol/L, high-density lipoprotein (HDL) cholesterol was 1.12 ± 0.26 mmol/L, total cholesterol (TC) was 4.39 ± 1.17 mmol/L, and homocysteine (HCY) was 15.15 ± 6.39 μmol/L. The complete baseline characteristic is shown in Table 1, which is divided into stroke and non-stroke categories. The clinical characteristics were balanced between the testing set and the training set, with no statistically significant differences in laboratory parameters or clinical risk factors, as detailed in Table 2.

**Table 1:** Comparison of baseline characteristics between the stroke and non-stroke groups.

Variables	Total (*n* = 666)	Testing set	Training set	Statistic	*p*
		(*n* = 134)	(*n* = 532)		
Age, yr	66.15 ± 9.87	67.60 ± 10.53	65.78 ± 9.67	*t* = 1.91	0.056
BMI	25.38 ± 3.21	25.03 ± 3.26	25.47 ± 3.19	*t* = −1.44	0.151
LDL, mmol/L	2.37 ± 0.85	2.32 ± 0.90	2.38 ± 0.84	*t* = −0.71	0.478
HDL, mmol/L	1.12 ± 0.26	1.13 ± 0.27	1.12 ± 0.26	*t* = 0.36	0.717
TC, mmol/L	4.39 ± 1.17	4.23 ± 1.02	4.43 ± 1.20	*t* = −1.93	0.055
TG, mmol/L	1.55 ± 1.17	1.49 ± 0.83	1.56 ± 1.24	*t* = −0.65	0.516
HCY, μmol/L	15.15 ± 6.39	15.41 ± 6.76	15.08 ± 6.30	*t* = 0.54	0.590
HbA1c	6.80 ± 1.80	6.89 ± 1.91	6.77 ± 1.77	*t* = 0.67	0.505
Sex (Male: 1; Female: 0), *n*(%)				*χ*^2^ = 0.25	0.619
0	266 (39.94)	51 (38.06)	215 (40.41)		
1	400 (60.06)	83 (61.94)	317 (59.59)		
Smoking (Yes: 1; No: 0), *n*(%)				*χ*^2^ = 1.66	0.198
0	446 (66.97)	96 (71.64)	350 (65.79)		
1	220 (33.03)	38 (28.36)	182 (34.21)		
Coronary heart disease (Yes: 1; No: 0), *n*(%)				*χ*^2^ = 0.10	0.747
0	376 (56.46)	74 (55.22)	302 (56.77)		
1	290 (43.54)	60 (44.78)	230 (43.23)		
Atrial Fibrillation (Yes: 1; No: 0), *n*(%)				*χ*^2^ = 1.64	0.200
0	631 (94.74)	124 (92.54)	507 (95.30)		
1	35 (5.26)	10 (7.46)	25 (4.70)		
Diabetes mellitus (Yes: 1; No: 0), *n*(%)				*χ*^2^ = 0.03	0.859
0	453 (68.02)	92 (68.66)	361 (67.86)		
1	213 (31.98)	42 (31.34)	171 (32.14)		
Hypertension (140/90 mmHg) (Yes: 1; No: 0), *n*(%)				*χ*^2^ = 0.51	0.473
0	280 (42.04)	60 (44.78)	220 (41.35)		
1	386 (57.96)	74 (55.22)	312 (58.65)		
Drinking (Yes: 1; No: 0), *n*(%)				*χ*^2^ = 0.71	0.399
0	566 (84.98)	117 (87.31)	449 (84.40)		
1	100 (15.02)	17 (12.69)	83 (15.60)		
History of cerebral infarction (Yes: 1; No: 0), *n*(%)				*χ*^2^ = 0.38	0.537
0	407 (61.11)	85 (63.43)	322 (60.53)		
1	259 (38.89)	49 (36.57)	210 (39.47)		

*t*, *t*-test; χ^2^, Chi-square test; SD, standard deviation.

**Table 2:** Baseline characteristics of the training and testing sets.

Variables	Total (*n* = 666)	Non-stroke group	Stroke group	Statistic	*p*
		(*n* = 367)	(*n* = 299)		
Age, yr	66.15 ± 9.87	64.86 ± 10.07	67.73 ± 9.39	*t* = −3.76	**<0.001**
BMI	25.38 ± 3.21	25.24 ± 3.03	25.55 ± 3.40	*t* = −1.22	0.223
LDL, mmol/L	2.37 ± 0.85	2.29 ± 0.78	2.47 ± 0.93	*t* = −2.68	**0.008**
HDL, mmol/L	1.12 ± 0.26	1.15 ± 0.26	1.08 ± 0.25	*t* = 3.04	**0.002**
TC, mmol/L	4.39 ± 1.17	4.33 ± 1.12	4.46 ± 1.23	*t* = −1.48	0.140
TG, mmol/L	1.55 ± 1.17	1.54 ± 1.22	1.56 ± 1.10	*t* = −0.21	0.834
HCY, μmol/L	15.15 ± 6.39	14.11 ± 5.21	16.42 ± 7.41	*t* = −4.55	**<0.001**
HbA1c	6.80 ± 1.80	6.82 ± 1.91	6.77 ± 1.66	*t* = 0.40	0.691
Sex (Male: 1; Female: 0), *n* (%)				*χ*^2^ = 1.04	0.307
0	266 (39.94)	153 (41.69)	113 (37.79)		
1	400 (60.06)	214 (58.31)	186 (62.21)		
Smoking (Yes: 1; No: 0), *n*(%)				*χ*^2^ = 7.23	**0.007**
0	446 (66.97)	262 (71.39)	184 (61.54)		
1	220 (33.03)	105 (28.61)	115 (38.46)		
Coronary heart disease (Yes: 1; No: 0), *n*(%)				*χ*^2^ = 57.35	**<0.001**
0	376 (56.46)	159 (43.32)	217 (72.58)		
1	290 (43.54)	208 (56.68)	82 (27.42)		
Atrial Fibrillation (Yes: 1; No: 0), *n*(%)				*χ*^2^ = 5.49	**0.019**
0	631 (94.74)	341 (92.92)	290 (96.99)		
1	35 (5.26)	26 (7.08)	9 (3.01)		
Diabetes Mellitus (Yes: 1; No: 0), *n*(%)				*χ*^2^ = 0.60	0.440
0	453 (68.02)	245 (66.76)	208 (69.57)		
1	213 (31.98)	122 (33.24)	91 (30.43)		
Hypertension (140/90 mmHg) (Yes: 1; No: 0), *n*(%)				*χ*^2^ = 14.00	**<0.001**
0	280 (42.04)	178 (48.50)	102 (34.11)		
1	386 (57.96)	189 (51.50)	197 (65.89)		
Drinking (Yes: 1; No: 0), *n*(%)				*χ*^2^ = 23.24	**<0.001**
0	566 (84.98)	334 (91.01)	232 (77.59)		
1	100 (15.02)	33 (8.99)	67 (22.41)		
History of cerebral infarction (Yes: 1; No: 0), *n*(%)				*χ*^2^ = 21.07	**<0.001**
0	407 (61.11)	253 (68.94)	154 (51.51)		
1	259 (38.89)	114 (31.06)	145 (48.49)		

*t*, *t*-test; *χ*^2^, Chi-square test; SD, standard deviation.

### Diagnostic performance of DL models

**Figure 4:**
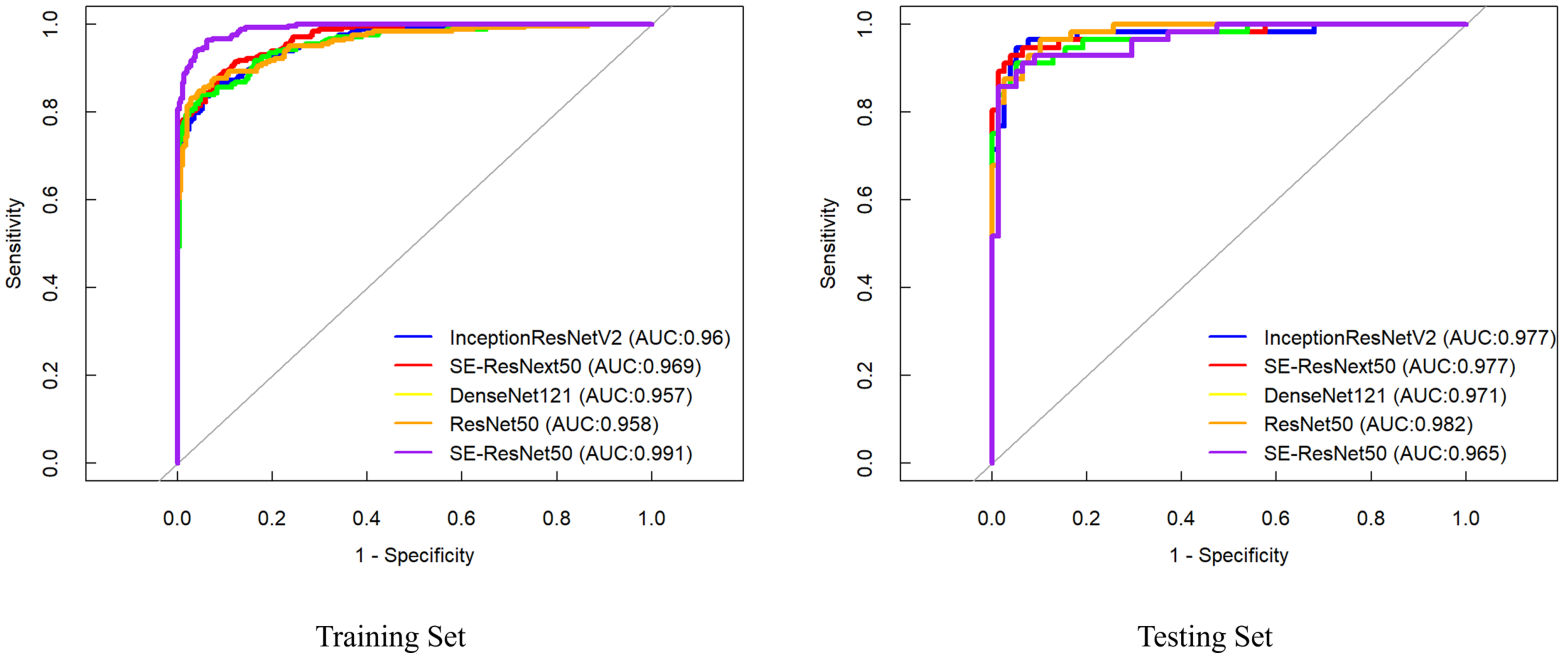
ROC curves of the DL models for predicting stroke risk based on carotid plaque ultrasound images.

All five architectures—ResNet-50, SE-ResNet-50, SE-ResNeXt-50, DenseNet-121, and InceptionResNet-V2—demonstrated robust diagnostic performance across both training and testing datasets (Figure 4). In the independent test set, the average inference time per image for all five deep learning models is less than 15 ms, ResNet-50 exhibited marginally superior overall metrics numerically, though DeLong’s test confirmed no statistically significant differences compared to other models. Notably, SE-ResNeXt-50 (accuracy = 94.8%) and InceptionResNet-V2 (accuracy = 94.8%) achieved marginally higher prediction accuracy for stroke risk stratification. SE-ResNeXt-50 demonstrated exceptional sensitivity (96.2%) in detecting stroke-prone cases, while ResNet-50 showed outstanding specificity (96.4%) in ruling out low-risk individuals (Table 3).

**Table 3:** Comparison of DL models based on carotid plaque ultrasound images for stroke risk prediction performance.

Deep learning model	Group	AUC (95% CI)	Accuracy	Sensitivity	Specificity	PPV	NPV
ResNet50	Training set	0.958 (0.941, 0.974)	0.908	0.831	0.972	0.962	0.873
	Testing set	0.982 (0.966, 0.998)	0.925	0.964	0.897	0.871	0.972
SE-ResNet50	Training set	0.991 (0.986, 0.996)	0.951	0.942	0.958	0.950	0.952
	Testing set	0.965 (0.937, 0.994)	0.925	0.911	0.936	0.911	0.936
SE-ResNeXt50	Training set	0.969 (0.958, 0.981)	0.898	0.893	0.903	0.886	0.909
	Testing set	0.977 (0.952, 1.000)	0.948	0.929	0.962	0.945	0.949
DenseNet121	Training set	0.957 (0.941, 0.973)	0.900	0.840	0.952	0.936	0.876
	Testing set	0.971 (0.945, 0.997)	0.933	0.911	0.949	0.927	0.937
InceptionResNetV2	Training set	0.960 (0.946, 0.974)	0.897	0.856	0.931	0.912	0.885
	Testing set	0.977 (0.950, 1.000)	0.948	0.946	0.949	0.930	0.961

### Diagnostic performance of ML models

**Figure 5:**
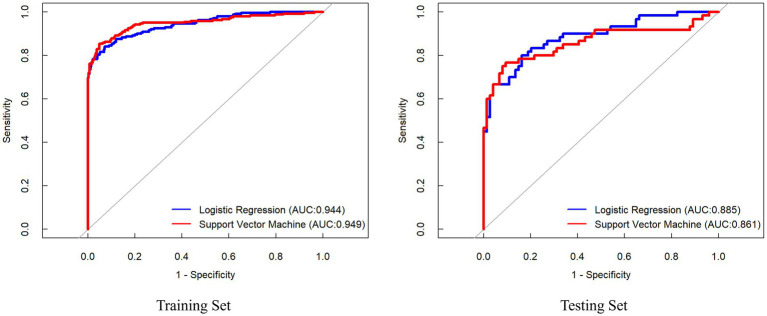
ROC curves of the ML models for predicting stroke risk based on carotid plaque ultrasound images.

In the training set, the SVM achieved the highest AUC (0.949, 95% CI: 0.929–0.970) with a sensitivity of 85.4% and specificity of 90.8% (Figure 5). However, in the independent test set, the LR model demonstrated superior diagnostic performance, attaining an AUC of (0.885 95% CI: 0.827–0.943), sensitivity of 80.8%, and specificity of 82.1%. Consequently, LR exhibited marginally better overall diagnostic efficacy than SVM, though DeLong’s test confirmed no statistically significant difference between the models (*z* = 0.591, *p* = 0.554) (Table 4).

**Table 4:** Comparison of ML models based on carotid plaque ultrasound images for stroke risk prediction performance.

Radiomics model	Group	AUC (95% CI)	Accuracy	Sensitivity	Specificity	PPV	NPV
Logistic regression	Training set	0.944 (0.924, 0.963)	0.887	0.841	0.925	0.901	0.877
	Testing set	0.885 (0.827, 0.943)	0.821	0.800	0.838	0.800	0.838
Support vector machine	Training set	0.949 (0.929, 0.970)	0.908	0.854	0.952	0.936	0.889
	Testing set	0.861 (0.790, 0.932)	0.836	0.767	0.892	0.852	0.825

### Comparison of the diagnostic performance between DL model and ML model

In the training set, the best DL model improved by 1.4% (0.958 vs. 0.944) over the best ML model. Similarly, in the testing set, the best DL model improved by 9.7% (0.982 vs. 0.885) over the best ML model. Indicating superior diagnostic performance. Beyond the AUC, other diagnostic metrics, including accuracy, sensitivity, and specificity, was consistently better for the DL model compared to the ML. This underscores the augmented capability of the deep learning model in predicting stroke risk among patients with carotid plaques, offering a more robust and reliable diagnostic tool.

## Discussion

In recent years, the global incidence and prevalence of ischemic stroke have exhibited a concerning upward trajectory. The pathogenesis of this condition involves multifaceted risk determinants, encompassing both non-modifiable factors—such as geographical disparities, sex, age, and ethnicity—and modifiable contributors including carotid artery stenosis, hypertension, diabetes mellitus, dyslipidemia, and adverse lifestyle behaviors (e.g., excessive alcohol consumption and smoking). Among these, carotid atherosclerosis constitutes the predominant modifiable clinical risk factor (24, 25). The CARE-II (China Atherosclerosis Risk Evaluation-II) study revealed a critical epidemiological pattern: among patients with cardiovascular and cerebrovascular diseases, high-risk carotid atherosclerotic plaques were 1.5-fold more prevalent than severe internal carotid artery stenosis (defined as ≥50% luminal narrowing). Notably, most individuals harboring vulnerable carotid plaques presented with stenosis degrees below 50% (26). Consequently, objective and precise characterization of carotid plaque attributes and their dynamic progression is paramount for effective ischemic stroke prevention. This imperative has positioned advanced morphological and functional plaque profiling as a cornerstone of contemporary stroke risk assessment research.

However, translating this cornerstone profiling into clinical practice faces significant hurdles. Current assessment methods are constrained by visual resolution thresholds and inherent subjectivity in interpretation, hindering detailed characterization of plaque microstructure and composition (27, 28). To address these limitations, artificial intelligence (AI)-driven image analysis approaches have emerged, with ML and DL constituting the two core paradigms. ML relies on radiomics to extract predefined quantitative features (e.g., shape, texture) from images, offering the advantage of strong model interpretability; whereas DL employs deep neural networks to autonomously learn discriminative features directly relevant to diagnostic objectives through end-to-end learning (29–31).

Through rigorous benchmarking comparisons, this study demonstrated that DL models significantly outperformed traditional ML models in predictive performance. The DL model achieved a notable 9.7% improvement in AUC within the test set, along with consistent superiority across multiple diagnostic metrics including accuracy, sensitivity, and specificity. These results confirm that for stroke risk stratification tasks related to carotid atherosclerosis, the DL paradigm inherently possesses stronger feature recognition and predictive capability compared to the ML paradigm. This superior performance is attributed to its capacity to autonomously extract subtle subvisual features—such as specific textural patterns indicative of intraplaque hemorrhage—that are undetectable by human visual assessment or conventional radiomics, thereby substantially enhancing the identification accuracy of vulnerable plaques.

Among the five DL architectures evaluated, although ResNet-50 demonstrated marginally superior performance metrics numerically, no statistically significant differences were observed between models (*p* > 0.05). This convergence in performance may be attributed to architectural saturation effects and a performance ceiling imposed by data limitations. Specifically, the approximately 50-layer depth of ResNet-50 appears sufficient to capture critical features from ultrasound images, while the inherent noise and anatomical variability in ultrasound imaging likely constrained the potential advantages of more complex architectures (e.g., InceptionResNet-V2) in leveraging multi-scale feature extraction capabilities.

Despite its superior performance, the “black-box” nature of DL—where the decision-making process lacks intuitive human interpretability—remains a fundamental barrier to its routine clinical adoption. Future research must prioritize the integration of explainable AI techniques to uncover the rationale behind model predictions through visualization and other methods. This will be crucial for maintaining high diagnostic accuracy while building clinicians’ trust, ultimately paving the way for the safe and effective integration of this powerful technology into stroke risk stratification workflows.

In this study, preprocessing procedures including noise reduction, gain standardization, and artifact removal were applied to ultrasound images prior to model training, which effectively attenuated the interference caused by various image artifacts and enabled the model to achieve favorable anti-interference capability and robustness during training and validation. However, it should be objectively noted that domain shift induced by discrepancies in clinical equipment and scanning parameters may compromise the generalization performance of the model, which represents a potential limitation of the present study. Therefore, this study warrants further investigation with larger and more diverse prospective cohorts under standardized protocols. Importantly, external validation in diverse populations, such as multi-center cohorts or across varying ultrasound devices, is currently absent and represents a critical limitation. Future steps should include multi-center validation and testing across different imaging devices to ensure the model’s adaptability and reliability in various clinical settings. Second, the developed DL model analyzed only ultrasound images and did not integrate clinical data. Therefore, in the next phase, we plan to combine clinical data with deep learning models to design and construct a novel hybrid model. In the future work section, we have expanded and refined the specific multimodal fusion strategy, which mainly includes feature-level concatenation of features extracted by CNN and clinical variables, as well as the adoption of a dual-branch network structure. Meanwhile, we also discuss the expected advantages of the proposed method, such as improving model calibration, enhancing robustness across different populations, and achieving more clinically interpretable risk stratification. Further improving its prognostic accuracy and risk stratification performance for stroke in patients with carotid atherosclerotic plaques.

### Limitations of the study

The sample size is limited, and it is necessary to expand the prospective cohort and conduct in-depth exploration under standardized protocols. And external validation across multiple centers and devices is not carried out, which further limits the generalization of the research conclusions. Based on this, in the future, we will integrate clinical data to construct a hybrid model to improve the clinical applicability of the model and its efficacy in predicting stroke risk.

## Conclusion

In summary, this study establishes that deep learning models—particularly the computationally efficient ResNet-50 architecture—significantly outperform conventional machine learning approaches in automated stroke risk stratification using carotid plaque ultrasound imaging (AUC improvement: 9.7%; *p* < 0.05). Crucially, the absence of statistically significant performance differences among five state-of-the-art CNN architectures (ResNet-50 vs. SE-ResNet-50/SE-ResNeXt-50/DenseNet-121/InceptionResNet-V2; *p* > 0.05) indicates architectural convergence under current data constraints, revealing a practical performance plateau rather than a bottleneck. This convergence underscores that clinical translation efforts should prioritize operational efficiency over marginal AUC gains. Consequently, ResNet-50 emerges as the optimal balance of predictive accuracy and deployability for real-time, resource-conscious clinical deployment—a critical advancement toward accessible stroke prevention in diverse healthcare settings.

## Data Availability

The original contributions presented in the study are included in the article/supplementary material, further inquiries can be directed to the corresponding author.

## Footnotes

1
http://premium.darwin.yizhunai.com

2
http://premium.darwin.yizhun-ai.com
